# Statistical errors and reporting deficiencies in clinical prosthodontic publications, 2019–2024

**DOI:** 10.1111/jopr.70086

**Published:** 2026-01-12

**Authors:** Ahmed Ben Suleiman, Charles F. Shuler, Ahmed Hieawy, HsingChi von Bergmann

**Affiliations:** ^1^ Department of Oral Health Sciences Faculty of Dentistry University of British Columbia Vancouver British Columbia Canada; ^2^ Department of Oral Biological and Medical Sciences Faculty of Dentistry University of British Columbia Vancouver British Columbia Canada

**Keywords:** assumption testing, dental research, prosthodontics, research methodology, statistical errors, statistical rigor, study design

## Abstract

**Purpose:**

Evidence‐based dentistry relies on valid statistics, yet misuse remains common. Despite increased emphasis on statistical rigor, recent prosthodontic publications remain underexplored. We hypothesized that statistical misapplications and reporting deficiencies persist in prosthodontic research. This study evaluated the appropriateness, application, and reporting of statistical tests in high‐impact prosthodontic journals.

**Methods:**

Original clinical studies published between January 2019 and December 2024 in the *Journal of Prosthodontics* (JOP), *International Journal of Prosthodontics* (IJOP), and *Journal of Prosthetic Dentistry* (JPD) were screened. Eligible articles used quantitative methods with inferential statistics. A 20% stratified random sample ensured representation by journal and year. Articles were assessed using a specialty‐adapted checklist covering five domains: test selection, sample size justification, assumption testing, examiner calibration, and result interpretation. A weighted scoring system categorized studies as having no major, minor, or major error. Descriptive statistics and Pearson's Chi‐square test, with Monte Carlo simulation as needed, assessed associations. Twenty percent of articles (*n* = 24) were double‐scored; consensus yielded inter‐rater reliability *κ* = 0.94.

**Results:**

A total of 119 clinical studies were reviewed (*JPD* 57, *IJOP* 37, *JOP* 25). Appropriate statistical tests were used in 88% of studies; notably, all 19 studies that involved a statistician demonstrated correct test selection. However, only 32% reported sample‐size calculations. Assumption testing was completed in 47% of studies, and examiner calibration was described in 40%. Confidence intervals were absent in 83% of reports, with only 8% both reporting and interpreting them. Overall, 45% of studies had no major statistical error, 35% contained major errors, and 21% showed minor deficiencies. Having no major error was strongly associated with proper test selection and assumption verification (*p* < 0.001).

**Conclusions:**

Statistical reporting deficiencies persist in prosthodontic research, particularly in sample‐size justification, assumption testing, and confidence interval reporting, highlighting the need for specialty‐specific checklists, enhanced training, and stronger editorial/reviewer standards.

Evidence‐based dentistry relies on sound statistical analysis to interpret clinical data appropriately, yet statistical misuse remains prevalent across dental disciplines.^1^ Such misuse undermines the reliability, reproducibility, and clinical applicability of dental research, echoing broader concerns about methodological rigor in biomedicine.^2^ In prosthodontics, where complex interventions, material variability, and long‐term outcomes are common, such flaws carry amplified consequences.^3^ Common issues include applying parametric tests to ordinal or non‐normal data, omitting assumption checks, failing to adjust for multiple comparisons, and inadequate reporting power or confidence intervals.^4^ These issues risk misrepresenting treatment effects, overstating material performance, and underestimating complications, thereby reducing scientific rigor and distorting clinical decisions.^5^ Although earlier studies, such as McGrath and Bedi,^5^ exemplified appropriate regression analysis and transparent confidence interval reporting, this level of rigor is not consistently observed across the prosthodontic literature. Statistical tests are not interchangeable; their validity depends on the data type and distribution. Parametric tests typically require continuous variables, approximately normally distributed variables with homogeneous variances, whereas non‐parametric approaches are better suited to ordinal data or situations where distributional assumptions are violated.^6^ Treating all numerical values as equivalent, such as using *t*‐tests on ordinal ratings, can mislead and overstate accuracy.^7^ Accordingly, careful alignment of test selection with data properties is essential and guided our evaluation of test appropriateness in this study.

Previous reviews across dental specialties have documented widespread statistical errors.^8^, ^9^ Kim et al. reported that more than half of dental articles contained at least one statistical error, often due to inappropriate test selection.^8^ Lucena et al. found that such misapplications altered study conclusions in 19% of cases.^9^ Additional concerns include multicollinearity, omission of confidence intervals, and inadequate justification for sample sizes.^10^, ^11^ Despite advances in reporting standards, prosthodontics has not been systematically assessed in recent years. This study is the first to systematically examine prosthodontic literature from 2019 to 2024, a period marked by growing emphasis on open science, expanded biostatistics training, and stronger editorial standards.^8^

This study addresses that gap by systematically evaluating the appropriateness, use, and reporting of statistical tests in prosthodontic research published from 2019 to 2024. We assessed test types and frequencies, identified common reporting deficiencies, and examined methodological practices in power calculations, normality assessments, and reliability testing. Given the absence of prior field‐specific reviews of this period, we adopted a descriptive approach without presupposing improvement or decline, allowing the data to characterize the current state of statistical reporting.

## MATERIALS AND METHODS

Articles were retrieved from the online archives of three peer‐reviewed prosthodontic journals: *Journal of Prosthodontics* (JOP), *International Journal of Prosthodontics* (IJOP), and *Journal of Prosthetic Dentistry* (JPD). These journals were chosen to isolate trends within prosthodontics, minimize cross‐specialty variation, and enable clearer benchmarking for future inter‐specialty comparisons.

Eligible articles were original clinical studies using quantitative methods with inferential statistical analysis, published between January 2019 and December 2024 in the target journals, and not retracted at the time of analysis. Exclusions included editorials, letters to the editor, technique articles, case reports, narrative or systematic reviews, in vitro studies, qualitative studies, and clinical studies without inferential statistics.

Articles from all three journals were exported into EndNote, with full texts screened twice by the primary investigator (A.BS.) using predefined criteria. Eligible articles were recorded in Microsoft Excel for structured data management.

**FIGURE 1:**
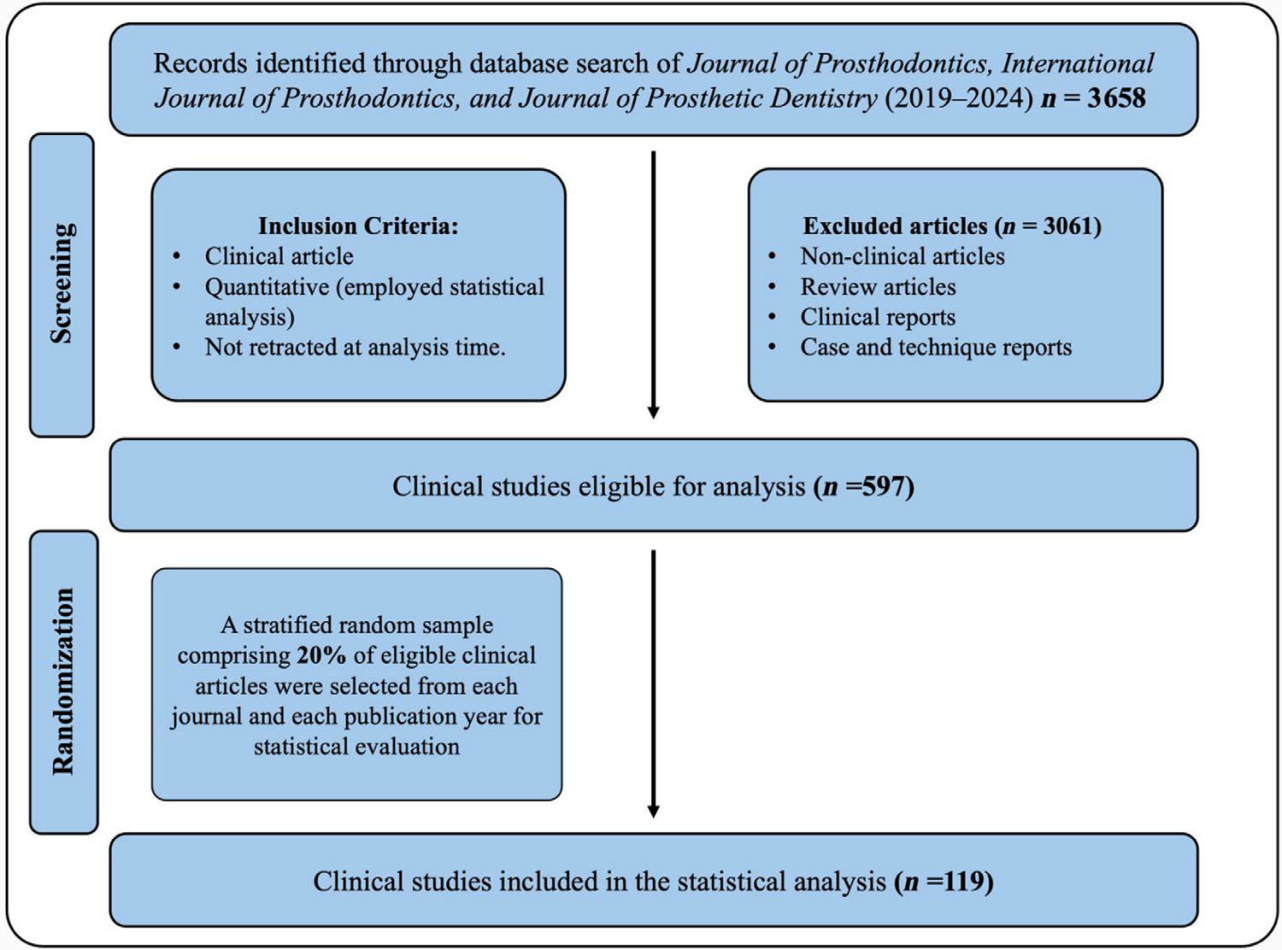
Screening and sampling flowchart for clinical articles in prosthodontic journals (2019–2024).

To ensure feasibility while maintaining representativeness, a 20% random sample of eligible articles was selected for detailed analysis (Figure 1). Random numbers were generated using Microsoft Excel's RANDBETWEEN function. To preserve proportional representation across journals and publication years, eligible articles were stratified by journal‐year, sorted by ascending random order, and the top 20% within the stratum were selected.

Sampling precision was estimated using the standard margin of error formula: MOE = 1.96 × √[*p*(1 − *p*)/*n*], assuming maximum variability (*p* = 0.5) and a sample size of *n* = 119. This yielded an MOE of 8.2% at 95% confidence, which is within the commonly accepted threshold (≤10%) for descriptive research, supporting the adequacy of the sample for estimating reporting practices.^12^

A checklist developed by the primary investigator (A.BS.), informed by applied statistical literature and prior evaluations in health and dental research,^8^, ^13^ and reviewed by a UBC faculty member (H.vB.), was used to assess reporting and methodological quality (Table 1). Articles were scored across five domains: test appropriateness, sample size justification, assumption testing, examiner calibration, and interpretation of results. Each domain was assessed independently to ensure consistent application of criteria and reduce cross‐domain bias.

**TABLE 1:** Statistical reporting and methodology checklist for clinical prosthodontic studies.

Domain	Evaluation objective	Key questions	Rating criteria	Weight
1. Appropriateness of statistical test selection	Ensure tests match study design, variables, and comparisons.	Are statistical tests appropriate for the study design, variables, and comparisons? If multiple or post hoc tests are used, are they justified and suitable?	Appropriate—All tests aligned with study design and objectives (scored 1). Not appropriate—At least one test mismatched without justification (scored 0).	0.35
2. Sample size calculation and justification	Assess whether sample size determination is transparent and appropriate.	Is sample size or power calculation reported? Is the method appropriate for the study design? If not calculated, is justification provided?	Justified—Sample size or power calculation reported and appropriate (scored 1). Not justified—Not reported or insufficiently justified (scored 0).	0.15
3. Statistical assumption testing (independence, normality, homogeneity)	Verify whether assumptions were identified, tested, and handled appropriately.	Is the experimental unit clearly defined (e.g., patient, tooth, crown, implant)? Were assumptions tested (independence, normality, variance homogeneity)? If independence was violated, was an appropriate method applied (e.g., GEE, mixed models)? Were results of assumption testing reported and interpreted?	Adequately tested—Independence met (1), normality and homogeneity tested (1 each). Inadequate—Key assumptions violated or untested (0). Partially tested—Some assumptions addressed or unclear (0.5).	0.35
4. Calibration and reliability	Assess reporting of measurement reliability.	Was examiner, instrument, or measurement reliability assessed? Was the method appropriate (e.g., ICC, kappa, repeatability)?	Reliable—Examiner calibration reported and appropriate (scored 1). Unreliable—Not assessed or insufficiently reported (scored 0).	0.05
5. Interpretation of results	Evaluate transparency and alignment of interpretation with evidence.	Are *p*‐values, and confidence intervals reported? Are non‐significant results and limitations discussed? Are assumption violations acknowledged? Is interpretation consistent with statistical evidence?	Met—Score 3–5: Interpretation aligns with evidence and transparent. Partially met—Score 2 to < 3: Some issues in interpretation or transparency. Not met—Score < 2: Interpretation lacks alignment or is unclear.	0.10

*Note*: Classification rules.

• No major statistical error: Composite score ≥ 0.80, or both Domains 1 and 3 scored 1 with a composite ≥ 0.70.

• Major statistical error present: Domain 1 or 3 scored 0, or composite score < 0.60.

• Minor statistical error present: Domain 1 or 3 scored 0.5, or composite score between 0.60 and 0.79 without meeting the criteria for “no major error.”

### Assessment domains

1. Appropriateness of statistical test selection (Domain 1): assessed alignment between statistical tests, study design, variable types, and research objectives.
2. Sample size calculation and justification (Domain 2): evaluated whether studies reported a formal sample size calculation or rationale.
3. Statistical assumption testing (Domain 3): examined whether relevant assumptions were addressed for the statistical tests used.
4. Calibration and reliability (Domain 4): considered whether examiner calibration was reported and methodologically adequate.
5. Interpretation of results (Domain 5): assessed completeness and clarity in reporting and interpreting statistical findings.
6. Overall evaluation: A weighted scoring system was applied to classify studies as having no major statistical errors (tests appropriate and assumptions adequately addressed), minor error (incomplete reporting prevented a definitive validity judgemet), or major statistical error (clear test‐variable mismatches or unaddressed assumption violations). Greater weight was assigned to Domain 1 (test appropriateness) and Domain 3 (assumption testing), as errors in these domains directly threaten internal validity and inference accuracy, whereas other domains primarily affect transparency and reproducibility.^14^ This weighting was conceptually motivated to reflect the relative impact of each domain on the reliability of study conclusions, rather than derived from any statistical modeling. Scoring criteria and full domain explanations are provided in Table 1.

### Statistical analysis

Descriptive statistics summarized the findings. Chi‐square goodness‐of‐fit tests assessed data distribution. Pearson's Chi‐square test, with Monte Carlo simulation when appropriate, assessed the associations between article characteristics (e.g., journal, publication year, continent of corresponding author, statistician involvement, and Domain 1‐5) and scoring outcomes (no major error, minor error, major error). This analysis addressed our secondary objective of identifying methodological features linked to statistical reporting quality. A 20% subset of sampled articles (*n* = 24) was independently double‐rated using the checklist by two reviewers; discrepancies were resolved by consensus. Inter‐rater agreement Cohen's *κ* was 0.94. All analyses were performed in R (version 4.5.1; R Foundation for Statistical Computing, Vienna, Austria), with a two‐sided *α* set at 0.05.

## RESULTS

### Descriptive characteristics of included studies

After 20% stratified random sampling to ensure representation by journal and year, a total of 119 articles met the inclusion criteria for assessment (Figure 1). Of these, nearly half (57) were published in *JPD*, 37 in *IJOP*, and 25 in *JOP*, an uneven distribution skewed toward *JPD* (*p* = 0.001). Publication was relatively even across 2019–2024, with no significant year‐to‐year variation (*p* = 0.659). Across domains, 88.2% of studies appropriately matched statistical tests to variable types and study design, yet only 31.9% reported a sample‐size calculation. Assumption testing showed that 47.1% of studies met all required assumptions; independence was most often satisfied, whereas homogeneity was least frequently tested for parametric tests. Examiner calibration was reported in 40.3% of studies, and formal reliability assessment was less common. For interpretation and discussion, *p*‐values were universally reported; however, confidence intervals were absent in 83.2% of articles, and only 7.6% both reported and interpreted them (Table 2).

**TABLE 2:** Descriptive summary of methodological and statistical characteristics in clinical prosthodontic studies (2019–2024, *n* = 119).

Variables	Categories	Frequency (*n*)	Percentage (%)
I. Journal‐related information			
Journal	*Journal of Prosthodontics*	25	21.0%
	*International Journal of Prosthodontics*	37	31.1%
	*Journal of Prosthetic Dentistry*	57	47.9%
Year of publication	2019	22	18.5%
	2020	14	11.8%
	2021	18	15.1%
	2022	24	20.2%
	2023	22	18.5%
	2024	19	16.0%
Statistician involvement	No	100	84.0%
	Yes	19	16.0%
Continent of corresponding author	Asia	46	38.6%
	Europe	38	31.9%
	North America	18	15.1%
	South America	13	10.9%
	Africa	3	2.5%
	Australia	1	0.8%
II. Domain 1—Appropriateness of statistical test selection			
Type of dependent variable	Continuous	43	36.1%
	Categorical	19	16.0%
	Ordinal	11	9.2%
	Mixed (≥ 2 types)	46	38.7%
Type of independent variable	Continuous	2	1.7%
	Categorical	105	88.3%
	Ordinal	1	0.8%
	Mixed (≥ 2 types)	11	9.2%
Number of study groups	< 3 groups	55	46.2%
	≥ 3 groups	64	53.8%
Pattern of test usage	Single statistical test	24	20.2%
	Multiple statistical tests	95	79.8%
Statistical test‐variable type match	Inappropriate test‐variable match	14	11.8%
	Appropriate test‐variable match	105	88.2%
III. Domain 2—Sample size calculation and justification			
Sample size calculation method	Not reported	81	68.1%
	Reported	38	31.9%
IV. Domain 3—Assumption testing			
Assumptions required for primary test	Independence only	62	52.1%
	Independence, normality, and homogeneity	57	47.9%
Independence of observations evaluation	Violation of independence	30	25.2%
	Independence maintained	78	65.5%
	Independence unclear	11	9.3%
Normality assumption evaluation	Not required, but tested	17	14.3%
	Not required, not tested	45	37.8%
	Required, not tested	38	31.9%
	Required, tested, and satisfied	19	16.0%
Homogeneity of variance evaluation	Not required, but tested	1	0.8%
	Not required, not tested	61	51.3%
	Required, not tested	49	41.2%
	Required, tested, and satisfied	8	6.7%
Overall assumption testing status	Assumptions not met	31	26.0%
	All assumptions met	56	47.1%
	Assumptions partially met	32	26.9%
V. Domain 4—Calibration and reliability			
Calibration mentioned	Calibration not mentioned	71	59.7%
	Calibration mentioned	48	40.3%
Calibration quality evaluation	Appropriate examiner(s) calibration	33	68.7%
	Inappropriate calibration (e.g., Tool only)	15	31.3%
VI. Domain 5: Results interpretation and discussion			
*p*‐Values reported	Not reported	0	0.0%
	Reported	100	100.0%
Confidence intervals reported and interpreted	Not reported	99	83.2%
	Reported, not interpreted	11	9.2%
	Reported and interpreted	9	7.6%
Non‐significant results discussion	Brief discussion	59	49.6%
	Fully discussed	60	50.4%
Limitations discussed	No	3	2.5%
	Yes	116	97.5%
Assumption violations reported	No	77	64.7%
	Yes	42	35.3%
Domain 5 overall evaluation	Not met	2	1.7%
	Fully met	59	49.6%
	Partially met	58	48.7%

*Note*: A Chi‐square goodness‐of‐fit test indicated a significant imbalance in the distribution of studies across journals, *χ* ^2^(2, *N* = 119) = 13.18, *p* = 0.001, with the *Journal of Prosthetic Dentistry* contributing disproportionately more studies than expected. In contrast, publication year was not significantly associated with study frequency, *χ* ^2^(5, *N* = 119) = 3.27, *p* = 0.659, suggesting a relatively even distribution of studies across the 2019–2024 period.

Of the 119 studies, 19 reported statistician involvement, and all satisfied Domain 1. Between group (statistician‐involved vs not‐involved) differences in Domains 2–5 were small in magnitute. By corresponding‐author region, studies most frequently originated from Asia (*n* = 46), followed by Europe (*n* = 38), North America (*n* = 18), South America (*n* = 13), Africa (*n* = 3), and Australia (*n* = 1). Journal distribution did not differ significantly by continent of the corresponding author, suggesting similar international representation across the three journals.

### Findings across domains

**FIGURE 2:**
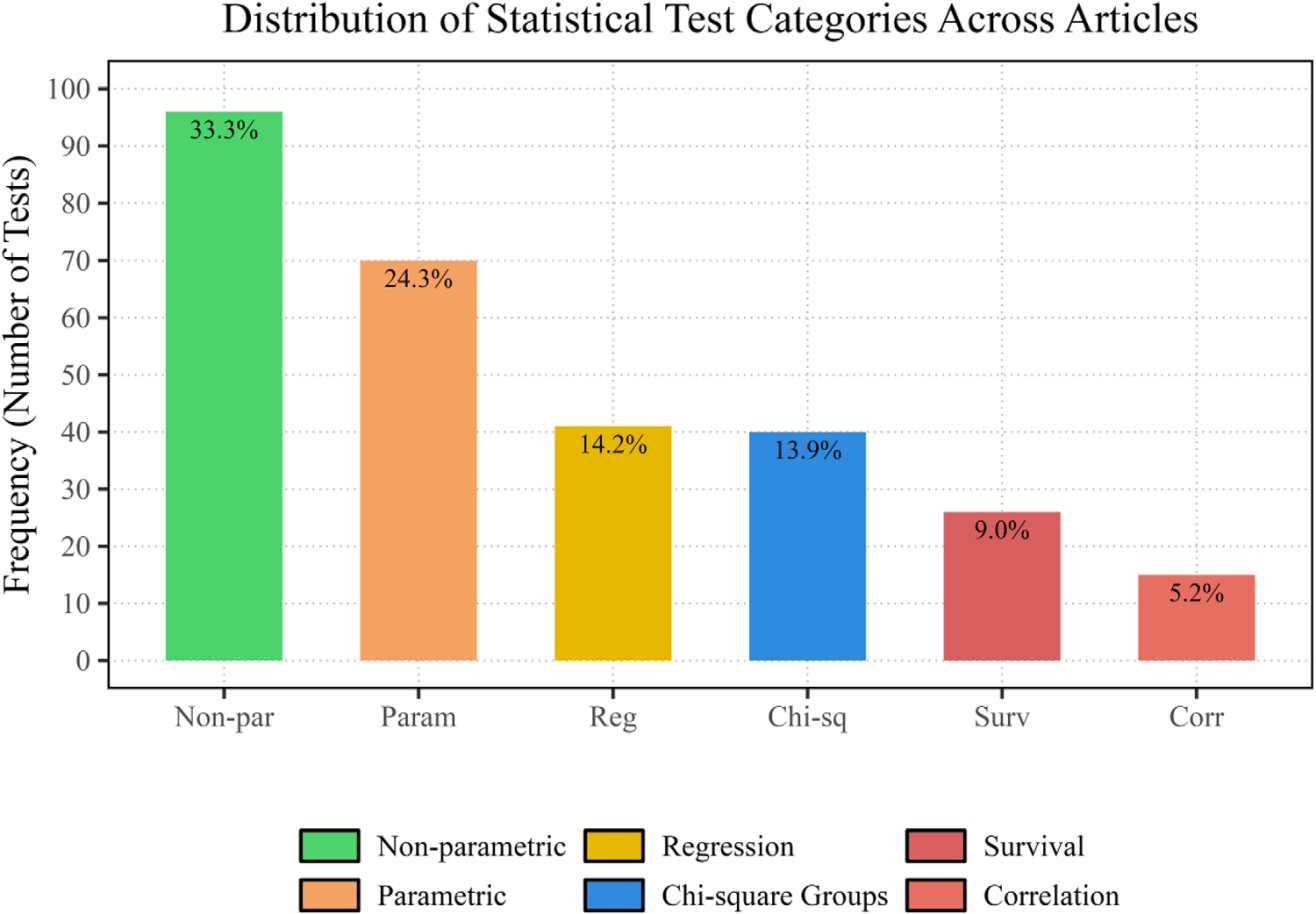
Distribution of statistical test types across 119 clinical prosthodontic studies (2019–2024). Nonparametric tests were most common (33%), followed by parametric tests (24%), regression analyses (14%), Chi‐square tests (14%), survival analyses (9%), and correlation methods (5%).

**FIGURE 3:**
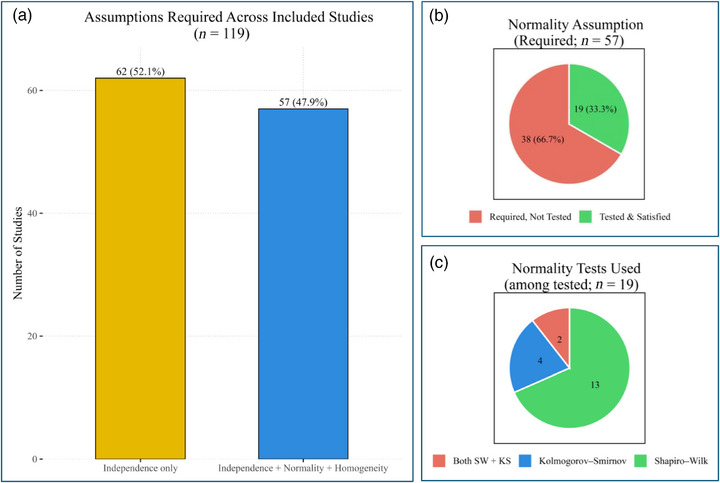
Assumption testing in 119 clinical prosthodontic studies (2019–2024). (a) Number of studies requiring only independence assumptions versus those requiring normality and homogeneity. (b) Proportion of studies that required assumption testing and reported conducting it. (c) Types of normality tests used, with Shapiro–Wilk most frequent (68%), followed by Kolmogorov–Smirnov (21%). Two‐thirds of studies requiring normality testing did not report performing it.

In Domain 1, nonparametric tests were most frequently used (33.3%), followed by parametric tests (24.3%), regression analyses (14.2%), Chi‐square groups (13.9%), survival analyses (9.0%), and correlation methods (5.2%) (Figure 2). In Domain 2, sample size calculation or justification was most commonly via power analysis (often via G*Power), effect size‐based estimation, or reference to prior studies. Fewer studies used pilot data or prevalence‐based formulae, and post hoc power analyses were rare. In Domain 3, reporting of assumption checks was inconsistent. Among studies requiring normality, two‐thirds did not report conducting a test. When normality was assessed, Shapiro–Wilk was most frequently applied (68.4%), followed by Kolmogorov–Smirnov (21.1%) and the combined use of both tests (10.5%) (Figure 3a–c). Among studies using parametric tests, 49 (41.2%) did not report homogeneity of variance, and only 8 (6.7%) assessed it exclusively with Levene's test, indicating substantial methodological gaps. In Domain 4, examiner calibration was reported in 40.3% of studies. The most common reliability metric was the intraclass correlation coefficient (ICC) (62.5%), followed by Kappa statistics (27.1%); Pearson correlation, Cronbach's alpha, and percentage agreement were less frequently used.

In Domain 5, P‐values were reported in all studies (100%). However, confidence intervals were omitted in 83.2% of studies, and only 7.6% both reported and interpreted them. Just over half of studies (50.4%) provided a full discussion of non‐significant findings, while nearly all (97.5%) acknowledged study limitations. Only 35.3% explicity reported assumption violations. Overall, 49.6% of studies fully met the criteria for Domain 5, while 48.7% met them partially, reflecting ongoing challenges in the communication and contexualization of statistical findings.

### Overall evaluation

**FIGURE 4:**
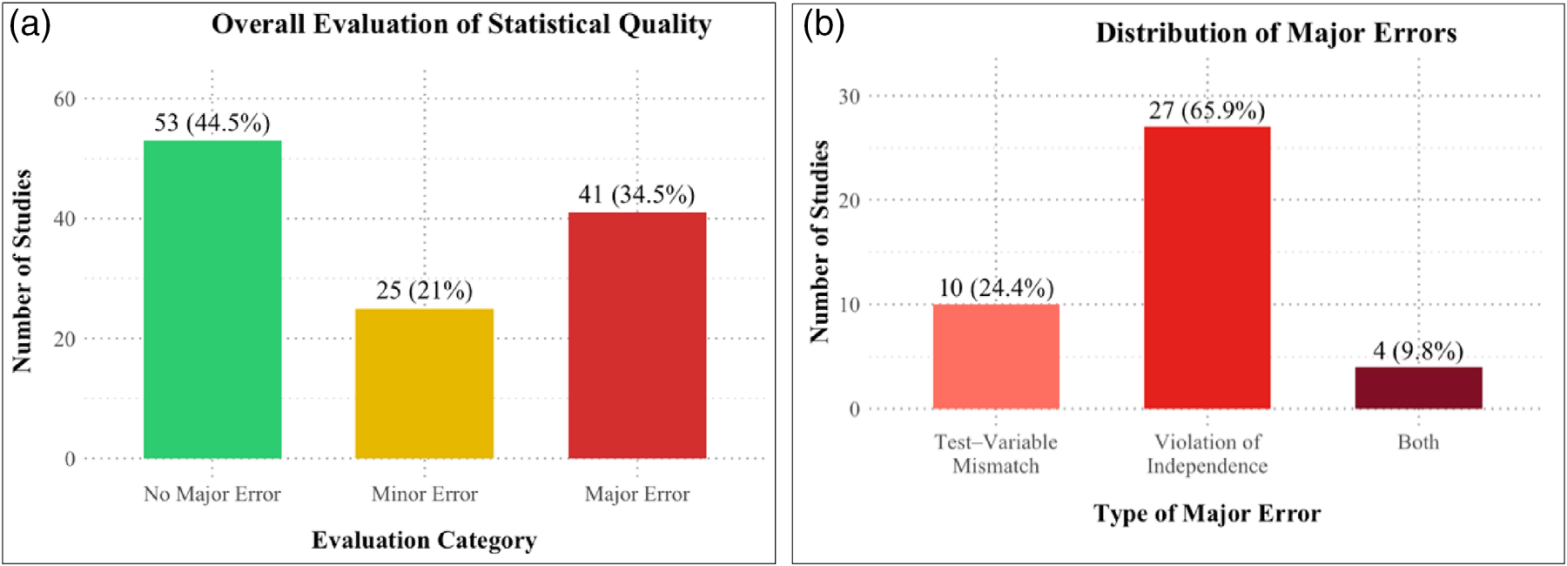
Overall evaluation and distribution of statistical errors in clinical prosthodontic studies (2019–2024). (a) Overall evaluation of statistical quality across included studies, with proportions classified as having no major error (44.5%), major error (34.5%), or minor error (21.0%). (b) Distribution of major errors, most of which involved violations of independence (65.9%), followed by inappropriate test–variable mismatches (24.4%), and concurrent violations of both (9.8%).

Overall, 53 studies were classified as having no major error, with statistical tests appropriately matched to variables, assumptions adequately addressed, and reporting transparent. Major errors were identified in 41 studies, most frequently due to inappropriate test–variable mismatches (24.4%, Domain 1), violations of independence assumptions (65.9%, Domain 3), or combined violations of both Domains 1 and 3 (9.8%) (Figure 4b). The remaining 25 studies exhibited minor errors, where incomplete reporting precluded a definitive assessment of statistical validity (Figure 4a). Most study characteristics showed no significant relationship with evaluation outcomes (*p* > 0.05). Significant associations were found for correct test‐variable matching and for meeting statistical assumptions (*p* < 0.001), both strongly linked to higher evaluation quality (Table 3).

**TABLE 3:** Associations between checklist domains and evaluation outcomes in clinical prosthodontic studies (2019–2024, *n* = 119).

Variable	*p*‐Value	Cramér's *V* (95% CI)	Effect size	Interpretation
Journals of publication	0.789	0.085 (0.060–0.240)	Negligible	No association
Year of publication	0.653†	0.183 (0.166–0.384)	Small	No association
Statistician involvement	0.824	0.057 (0.022–0.271)	Negligible	No association
Continent of corresponding author	0.720†	0.178 (0.158–0.341)	Small	No association
Domain 1: Match between statistical test and variable type	**<0.001** †, [Link]	0.504 (0.370–0.627)	Large	Strong association
Domain 2: Sample size calculation reported	0.345	0.134 (0.033–0.355)	Small	No association
Domain 3: Assumptions of statistical tests met	**<0.001** *	0.873 (0.808–0.937)	Large	Strong association
Domain 4: Calibration procedures reported	0.064	0.193 (0.116–0.320)	Small	Borderline significant
Domain 5: Reporting and interpretation of results	0.502†	0.120 (0.079–0.269)	Small	No association

*Note*: Effect size interpreted according to Cohen's criteria (small = 0.1, medium = 0.3, large = 0.5).

Abbreviation: CI, confidence intervals generated using nonparametric bootstrap (1000 replicates, percentile method). Bootstrapping is a Monte Carlo resampling method used to estimate uncertainty in effect size measures, such as Cramér's V.

^†^ Monte Carlo exact *p*‐values reported where > 20% of expected cell counts were < 5.

Bold * *p* < 0.05.

## DISCUSSION

This study is the first to systematically examine how statistical methods are reported and applied in clinical prosthodontics across several leading journals between 2019 and 2024. Unlike prior work in broader dental research or other specialties,^8^, ^9^, ^15^, ^16^ this study isolates patterns specific to prosthodontics, identifying discipline‐specific gaps and trends that have previously gone unrecognized.

The uneven distribution of articles across journals, driven by differences in overall publication volume during the study window, does not indicate selection bias. The wide geographic spread of corresponding authors underscores the global nature of prosthodontics research and suggests that methodological shortcomings were widespread rather than region‐specific.

Despite increased access to statistical training and the availability of reporting frameworks such as Consolidated Standards of Reporting Trials (CONSORT)^17^ and Strengthening the Reporting of Observational Studies in Epidemiology (STROBE),^18^ key deficiencies persist. Our findings echo earlier reports^8^, ^9^: essential steps, such as test selection, assumption checking, sample‐size justification, examiner calibration, and interpretation, are often insufficiently addressed. The absence of discipline‐specific guidance in author guidelines is likely a contributing factor.

Only 19 articles noted statisticians’ involvement. Although these consistently met Domain 1 (appropriate test‐data matching), such engagement alone may not secure overall quality when statisticians are involved narrowly (e.g., test selection or analysis only) rather than throughout study design, assumption verification, and manuscript preparation. A more integrated statistical role, combined with a standardized checklist tailored to prosthodontics, could promote consistency in test selection, assumption testing, and sample‐size justification.^2^, ^4^

Although Domains 1 and 3 were significantly associated with overall evaluation scores, as expected due to their weighting and direct impact on statistical validity, not all domains demonstrated such associations. For example, Domain 2 (sample size justification) was not statistically linked to evaluation outcome, despite its conceptual importance. This may reflect widespread underreporting rather than a lack of sample planning, or editorial practices that do not consistently require justification. These findings underscore the distinction between what is methodologically essential and what is routinely enforced or reported in prosthodontic publications.

The consequences extend beyond technical details. Misapplied methods can inflate claims about treatment success or longevity.^2^ Overestimated survival rates may set unrealistic expectations for patients and clinicians,^19^ small or poorly justified samples can miss meaningful effects,^20^ and failure to account for clustering can make certain materials or designs appear unduly effective.^21^ Notably, when studies with inappropriate analyses were re‐evaluated, conclusions changed in roughly 19% of cases, highlighting how analytic flaws can reshape the interpretation of treatment outcomes.^9^ When statistical decisions are unsound, the evidence base that informs treatment choices, insurance coverage, and patient counselling becomes less reliable.^2^, ^11^

### Key areas of statistical concern identified

Threview highlighted four areas where problems were especially frequent.

1. Parametric tests applied to ordinal or categorical data: A common error was using *t*‐tests or ANOVA on ordinal data without transformation or checks of distributional assumptions,^6^, ^22^ creating a false sense of precision. Researchers often defaulted to familiar methods without verifying suitability. Clearer guidance at the design stage would help prevent this.^7^
2. Ignoring data clustering: Many studies analyzed teeth, implants, or prostheses as independent units even when they belonged to the same patient,^23^ inflating statistical significance. Split‐mouth or bilateral designs were likewise mishandled. Clustering should be planned for and modelled, with an explicit description of how non‐independence was addressed.^24^
3. Small or poorly justified sample sizes: Some studies used very small samples (sometimes <10 per group) for complex analyses (e.g., survival, regression), resulting in low power and unstable estimates.^25^ Sample‐size justification was consistently underreported, regardless of sample size or publication year, with no association between reporting and either factor. When full recruitment is infeasible, studies should be labelled exploratory and interpreted cautiously.^4^ Transparent a priori justification remains essential to reliability and reproducibility.
4. Missing confidence intervals and weak interpretation: *p*‐Values were commonly reported, but confidence intervals were often absent or not discussed in clinically meaningful terms. Notably, their omission in 83.2% of studies limits the interpretability of effect sizes and reduces the clinical relevance of statistical findings. Even when present, links to clinical significance were rare. Reporting both *p*‐values and confidence intervals and interpreting them improved transparency and utility.^26^

Taken together, these findings suggest that awareness may be improving, yet errors remain widespread. Addressing them will require enhanced researcher training, specialty‐specific reporting checklists, a stronger emphasis on assumption testing, and stricter editorial standards.

### Strengths, limitations, and future directions

This study's strengths include its use of a structured scoring protocol, calibration between reviewers, and focus on recent literature. However, limitations include restriction to English‐language, high‐impact journals, limiting generalizability and trend analysis. While a full census was not feasible, the stratified 20% sample provided sufficient precision (MOE = 8.2%) to support descriptive inferences. Given the absence of historical prosthodontic benchmarks, the findings serve as a foundational dataset for future trend monitoring and cross‐specialty comparisons. Future research should evaluate statistical reporting in a broader range of journals and languages, track trends over time, and assess the impact of structured statistical checklists.

## CONCLUSION

Statistical deficiencies remain common in prosthodontic research, particularly in sample‐size justification, assumption testing, and confidence interval reporting. Nearly one‐third of studies exhibited major errors likely to compromise validity and interpretation. To improve methodological rigor and clinical relevance, greater involvement of statisticians, adoption of specialty‐specific checklists, and stricter editorial oversight are recommended.

## CONFLICT OF INTEREST STATEMENT

The authors declare no conflicts of interest and no relationship with the *Journal of Prosthodontics*, *the International Journal of Prosthodontics*, or *the Journal of Prosthetic Dentistry*.

## Data Availability

The data that support the findings of this study are available from the corresponding author upon reasonable request.
